# Book Review: Neanderthal Language: Demystifying the Linguistic Powers of Our Extinct Cousins

**DOI:** 10.3389/fpsyg.2021.702361

**Published:** 2021-05-28

**Authors:** Petar Gabrić

**Affiliations:** Institute for German Linguistics, Philipps University of Marburg, Marburg an der Lahn, Germany

**Keywords:** language evolution, Neanderthals, Paleolithic, Neanderthal language, cognitive archaeology

Recently, we have witnessed an explosion of studies and discussions claiming that Neanderthals engaged in a range of “symbolic” behaviors, including personal ornament use (Radovčić et al., 2015), funerary practices (Balzeau et al., 2020), visual arts (Hoffmann et al., 2018), body aesthetics (Roebroeks et al., 2012), etc. In Paleolithic archaeology, it has become mainstream to axiomatically infer from these putative behaviors that Neanderthals engaged in symbol use and that Neanderthals thus possessed some form of language. Rudolf Botha's bombastic title *Neanderthal Language: Demystifying the Linguistic Powers of Our Extinct Cousins* provides a detailed and very critical overview of the archaeological hypotheses and speculations about Neanderthal language.

Because language does not fossilize, eventual linguistic abilities of extinct hominins have to be inferred from indirect evidence. In the first two chapters, Botha introduces this “windows approach” to language evolution and proposes three conditions that should be met for window inferences to be sound. The inferences and conclusions must be *pertinent*—the phenomenon referred to (i.e., language) should be accurately identified, properly *grounded* in data about the window phenomenon, and *warranted*—the inferential step from the window phenomenon to language evolution should be justified. In chapters three to seven, Botha comprehensively reviews the current archaeological knowledge on the putatively “symbolic” Neanderthal behaviors, including putative jewelry, cave art, body aesthetics, and funerary practices. Botha masterfully demonstrates that there is no logical foundation to conclude that these putative behaviors reflect or constitute symbol use (in the Peircean or Saussurean sense), let alone language use. Botha (p. 54) asks: “What are the distinctive properties of symbols according to [these accounts]?” No adequate answer to this question is given in the archaeological literature. It is revealed that archaeologists typically axiomatically infer symbol use from the above-mentioned behaviors, e.g.: “[A]bstract or depictional representations and personal ornaments are the only unquestioned evidence of the emergence of symbolism.” (d'Errico, 2009, p. 108). According to Botha, even if these behaviors were symbolic, the inferential step from these symbolic behaviors to language would be an arbitrary one.

Chapters eight to ten are devoted to stone-tool-related behaviors and hunting as examples of “non-symbolic” Neanderthal behaviors. Botha criticizes the literature on the relationship between Paleolithic stone tools and language evolution, arguing among others that stone tool production is very different from language. On the other hand, Botha somewhat surprisingly proposes that ambush hunting of large prey may indicate that Neanderthals possessed some form of language. Botha believes that such behaviors required close cooperation between individuals and thus communication—possibly, linguistic communication. Based on these data, Botha concludes his book by proposing that Neanderthals had a “simpler” form of language.

Botha can be praised for his critical review and evaluation of the archaeological studies of “symbolic” Neanderthal behaviors through which he demonstrates that the association between the putatively “symbolic” behaviors and actual linguistic symbol use is completely arbitrary, i.e., not based on any linguistic ontology. Botha is not only critical but offers a tentative framework for the evaluation of the existing as well as future window inferences in language evolution.

On the other hand, Botha's account of the eventual relationship between stone-toolmaking-related behaviors and the linguistic status in Neanderthals is less convincing as well as misleading. Most problematically, Botha pays practically no attention to specifically Neanderthal stone tools but discusses older forms of Paleolithic stone tools. Botha rejects the putative hypothesis that stone toolmaking and language are so similar that the presence of stone toolmaking indicates the presence of language, citing Stout and his colleagues among others. However, Stout is not a proponent of such a hypothesis, but of a hypothesis that there are cognitive processes which underlie both stone-tool-related and linguistic behaviors, and that language thus might rely on processes and networks which were reused from processes and networks “originally” involved in stone-tool-related behaviors, e.g.: “[E]arly stone tool making [possibly] favored adaptations later incorporated into an evolving language capacity” (Stout and Chaminade, 2007, p. 1098; cf. Gabrić et al., 2018). Further, to support his assertion that stone toolmaking is not “recursive” (without defining what recursion is), Botha cites Berwick and Chomsky (2016) adding that the two are not sure whether the knapping procedure can be depicted as a “syntactic tree.” Whether some people think that something can or cannot be depicted as a tree should not guide conclusions about whether a phenomenon is “recursive” or not.

Botha's conclusions are also somewhat obscure. Botha (p. 156) proposes that Neanderthals possessed “a form of language with basic units resembling arbitrary linguistic signs” which “lacked the complex grammatical properties of full modern language.” It is unclear what Botha wants to say with this conclusion. What kind of language does not contain linguistic signs (but “units resembling” them)? That seems paradoxical. What are “complex grammatical properties” (and what are simple ones?) and why aren't they needed for ambush hunting of large prey? What are “full modern languages” and on which properties is the gradation of “language modernity” based? Did Neanderthal languages have syntactic constituents, word classes, transitivity, morphological processes, prosody, a large repertoire of consonants and vowels, etc.? Why does Botha not discuss previous and linguistically more detailed accounts of Neanderthal language (Progovac, 2016)? Unfortunately, Botha's window inference fails at (at least) one of his own principles for sound inferences—it is impertinent, i.e., the linguistic phenomenon in question is not adequately identified. Furthermore, his conclusions are based on a very brief review of studies on Neanderthal hunting, studies tentatively suggesting an association between the FOXP2 variants in Neanderthals and language and studies suggesting that specific, yet very broadly demarcated, brain areas are smaller in size or volume in Neanderthals compared to modern humans. Firstly, the FOXP2 hypothesis is not characterized in adequate detail (cf. Kuhlwilm, 2018), while a number of other topics in Neanderthal genetics have emerged throughout the years apart from the endlessly discussed FOXP2 (Murphy and Benítez-Burraco, 2018; Silvert et al., 2019; Villanea and Schraiber, 2019; Taskent et al., 2020; Reinscheid et al., 2021). Secondly, to suggest that the smaller size or volume of particular brain areas in Neanderthals compared to modern humans is indicative of both the presence and absence of specific linguistic features in Neanderthals (p. 156) is highly controversial at best. Thus, Botha's conclusions also appear ungrounded and unwarranted.

In conclusion, Botha's (2020) evaluation of the “symbolic” hypotheses about Neanderthal language is of great value, given that previous criticisms of such axiomatic thinking have not received adequate attention (Botha, 2009, 2010, 2012, 2015; Garofoli, 2014; Garofoli and Iliopoulos, 2019). In fact, chapters three to seven should be mandatory reading for anyone interested in studying Neanderthal behavior, both experts and non-experts. Further, Botha's proposal that specific hunting behaviors may be an indicator of linguistic communication in Neanderthals will hopefully induce future discussions on this hypothesis. On the other hand, Botha's account of the relationship between Neanderthal stone tools and language may be somewhat problematic, while his conclusions lack substance.

## Author Contributions

PG was solely responsible for this manuscript.

## Conflict of Interest

The author declares that the research was conducted in the absence of any commercial or financial relationships that could be construed as a potential conflict of interest.
